# Psychotropic Medication and Substance Use during Pregnancy by Women with Severe Mental Illness

**DOI:** 10.3389/fpsyt.2017.00028

**Published:** 2017-02-17

**Authors:** Kate J. Brameld, Assen Jablensky, Jenny Griffith, John Dean, Vera A. Morgan

**Affiliations:** ^1^Neuropsychiatric Epidemiology Research Unit, School of Psychiatry and Clinical Neurosciences, The University of Western Australia, Crawley, WA, Australia; ^2^Centre for Population Health Research, Faculty of Health Sciences, Curtin University, Perth, WA, Australia; ^3^Centre for Clinical Research in Neuropsychiatry, School of Psychiatry and Clinical Neurosciences, The University of Western Australia, Crawley, WA, Australia

**Keywords:** mental illness, pregnancy, demographic factors, hospitalization, psychotropic medication, medical record linkage

## Abstract

**Background:**

Sociodemographic factors, alcohol and drug intake, and maternal health are known to be associated with adverse outcomes in pregnancy for women with severe mental illness in addition to their use of psychotropic medication. In this study, we describe the demographic characteristics of women hospitalized for severe mental illness along with their use of medication and other drugs during the pregnancy period.

**Methods:**

A clinical case note review of women with psychosis who were hospitalized at the State Psychiatric Hospital in Western Australia during 1966–1996, gave birth between 1980 and 1992, and received psychiatric treatment during the pregnancy period. The mother’s clinical information was available from the case notes and the midwives record. The demographic characteristics of the mothers were described together with their hospitalization pattern and their medication and substance use during the pregnancy period.

**Results:**

A total of 428 mothers with a history of severe mental illness were identified who gave birth during 1980–1992. Of these, 164 mothers received psychiatric care during the pregnancy period. One hundred thirty-two had taken psychotropic medication during this period. Mothers who were married, of aboriginal status or living in regional and remote areas appeared less likely to be hospitalized during the pregnancy period, while older mothers and those with a diagnosis of schizophrenia were more likely to be hospitalized. The number of mothers taking psychotropic medication in the first trimester of pregnancy was reduced compared to the previous 6 months. The decline in the number taking substances over the same period was not significant. In all, 16% of the women attempted suicide during the pregnancy period and 10% non-suicidal self-injury.

**Conclusion:**

The women demonstrate a pattern of decreased use of psychotropic medication use from the period before pregnancy to the first trimester of pregnancy. Our data highlight the importance of women with severe mental illness receiving regular ongoing monitoring and support from their psychiatrist during pregnancy regarding the level of medication required as well as counseling with regard to substance use, non-suicidal self-injury, and attempted suicide.

## Introduction

Sociodemographic factors (1), alcohol and drug intake (2–4), use of psychotropic medication, and untreated disease, which may be exacerbated or recur during pregnancy, have been shown to be associated with adverse outcomes in pregnancy for women with severe mental illness (5–11). Here, we report on a small case-comparison study where data on medication and drug intake, attempted suicide, and non-suicidal self-injury was available for women who had received psychiatric care for severe mental illness during their pregnancy period. Our aims were to describe the demographic characteristics of women hospitalized for severe mental illness along with their pattern of hospitalization and their use of medication and other drugs during the pregnancy period. For the purposes of this study, the pregnancy period is defined as commencing 6 months prior to pregnancy until the end of pregnancy.

## Materials and Methods

Women with a diagnosis of schizophrenia, bipolar disorder, or unipolar major depression who were treated at the State Psychiatric Hospital of Western Australia between 1966 and 1996 and gave birth between 1980 and 1992 were identified *via* linkage of the WA Midwives Notification System and the WA Mental Health Information System (MHIS). Further information was collected by clinical case note review for the women who were treated by psychiatric inpatient or outpatient services during the pregnancy period. The study focuses on this subgroup of women.

The study had University of Western Australia Human Research Ethics Committee approval as well as specific approvals from the individual inpatient and outpatient mental health services at which the clinical records were held. The data were analyzed using SAS version 9.4 (SAS institute Inc., Cary, NC, USA).

The International Classification of Diseases, ninth Revision codes (12) were used to identify specific disorders: schizophrenia (295.0–295.9), bipolar disorder (296.0, 296.2–296.5), and unipolar major depression (296.1, 296.6, 296.8, 296.9). The concurrent validity of MHIS ICD-9 diagnoses had been ascertained against an independent case sample assessed using a semi-structured diagnostic interview (13), which established sensitivity of 0.92 and specificity of 0.88 for schizophrenia and 0.80 and 0.90, respectively, for affective disorders (14). The clinical case note review was undertaken by two of the coauthors (John Dean and Jenny Griffith) using structured checklists developed specifically for this study to collect information on medications, substance use, attempted suicide, non-suicidal self-injury, and other clinical and risk data for the women who were treated by psychiatric inpatient or outpatient services during the pregnancy period. If the women had attended other hospitals/outpatient services in addition to the State Psychiatric Hospital, medical records from these sites were also retrieved where possible. Women were recorded as having used psychotropic medication and/or substances according to the trimester of pregnancy or prepregnancy period, if this was recorded in their medical record while they were being treated, either as an inpatient or an outpatient. Variables were created for each trimester and for each group of psychotropic medications to indicate whether or not the women were recorded as using the medication during that period of time. Psychotropic medications recorded included typical antipsychotics, tricyclic antidepressants, mood stabilizers, benzodiazepines, and hypnotics. Substances recorded included alcohol, cannabis and other.

No further information was collected for the women who did not receive psychiatric inpatient or outpatient care during the study, but their demographic information was available for comparison with those who did receive psychiatric care.

Demographic and clinical information was compared for all mothers included in the study, those receiving psychiatric care during their pregnancy period and those receiving psychiatric care and who took medication during the pregnancy period. For comparison of demographic characteristics, socioeconomic status was measured using the Australian Bureau of Statistics Socioeconomic Indices for Areas—index of relative disadvantage (15) and accessibility/remoteness was measured using the ARIA + index (16). Variation in demographic characteristics of mothers for all births and for those admitted to hospital was conducted using a binomial test for differences between proportions.

The number of births where the mother was hospitalized voluntarily with a psychiatric diagnosis was calculated as was the average length of stay per birth in days for these admissions. This was repeated for involuntary admissions.

The number of mothers taking psychotropic medication and substances was calculated for the 6-month period before pregnancy and for each trimester. Whether or not there was a significant change in the proportion of women taking medication or substances prepregnancy and in the first trimester of pregnancy and then between the first and second trimester and the second and third trimester was tested using a *t*-test for paired samples.

## Results

**Figure 1:**
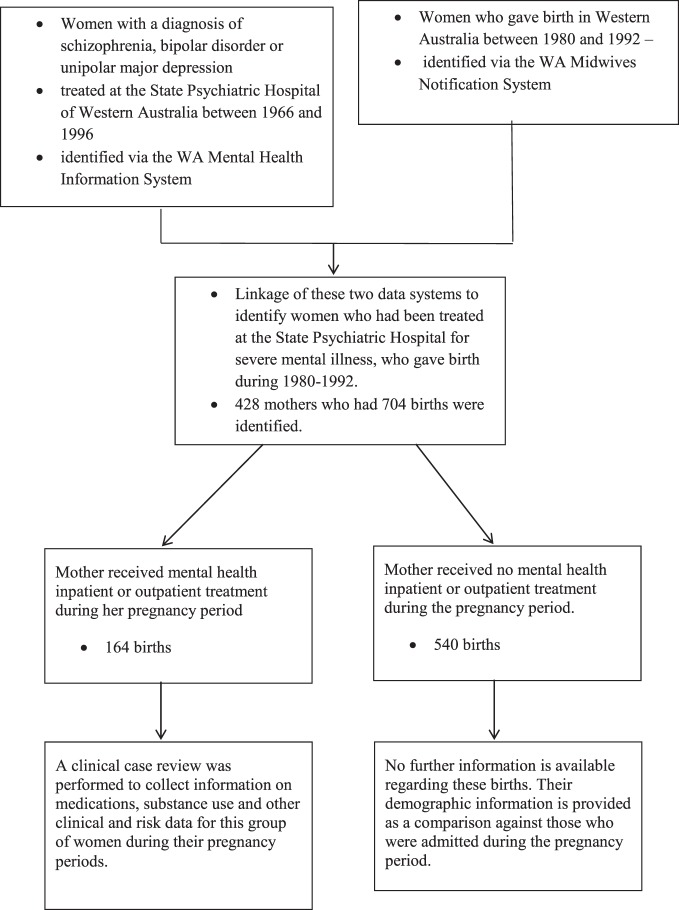
**Selection of patients in the study**.

The selection of the 428 mothers into the study is illustrated in Figure 1.

The demographic characteristics of the mother relating to each birth are shown in Table 1. This shows that of the three diagnostic groups included in the study, mothers with schizophrenia were most likely to receive psychiatric care during the pregnancy period and those with unipolar depression were least likely to receive treatment. A lower proportion of those who were married or in a *de facto* relationship were in the group who were hospitalized compared to those who were single, divorced, or separated. The proportions of aboriginal mothers and those living in regional and remote areas were also lower in the hospitalized group. Mothers aged over 34 years appeared more likely to be hospitalized than their younger counterparts, as were those living in the city. Parity and low socioeconomic status appeared to have little effect on the risk of hospitalization during the pregnancy period. Table 2 shows hospital admission data for each mother during the pregnancy period. This was a group of mothers at high risk for suicide and non-suicidal self-injury as indicated by 24 suicide attempts by 22 of the mothers (a suicide attempt in 14.6% of pregnancy periods) and 15 cases of non-suicidal self-injury by 14 mothers (9%) during the pregnancy period. Thirteen of the mothers had an intellectual disability (8%).

**Table 1:** **Demographic characteristics of mother for each birth**.^a^

Demographic variable	All births—status of mother		Births to mothers receiving psychiatric treatment during the pregnancy period—cases		Births to mothers not receiving psychiatric treatment during the pregnancy period—comparison		Births to mothers receiving psychiatric treatment and who took medication during the pregnancy period (subset of cases)	
				
	*N*	% of total	*N*	% of total	*N*	% of total	*N*	% of those admitted during pregnancy period
No. of births	704	100	164	23	540	77	132	80
No. of mothers	428	100	138	32	290	68	113	82
Parity								
1	428	61	104	63	324	60	87	66
2	207	29	47	29	160	30	35	27
3+	69	10	13	8	56	10	10	7
**Diagnosis**
Schizophrenia	293	42	84	51*	209	39	69	52
Bipolar disorder	317	45	68	42	249	46	56	43
Unipolar depression	94	13	12	7*	82	15	7	5
**Age group**
≤19 years	50	7	8	5	42	8	6	5
20–34 years	584	83	133	81	451	83	107	81
≥35 years	70	10	23	14*	47	9	19	14
**Marital status**
Married/*de facto*	505	72	99	60*	406	75	67	61
Single/divorced/separated	199	28	65	40*	134	25	46	39
Aboriginality	70	10	7	4*	63	12	5	4
SES (bottom 20th percentile)	216	31	52	32	164	31	46	35
**ARIA**
City	456	65	131	80*	325	61	108	82
Regional	151	31	22	13*	129	24	16	12
Remote	91	4	11	7	80	15	8	6

*^a^Mothers who gave birth during 1980–1992 and had also been an inpatient at the State Psychiatric Hospital during 1966–1996*.

*p < 0.05 significant difference between births exposed to and births not exposed to psychiatric treatment during the pregnancy period.

*SES, socioeconomic status measured as SEIFA, socioeconomic indices for areas (15)*.

*ARIA, accessibility remoteness index of Australia (16)*.

**Table 2:** **Mothers’ hospital admission profile during the pregnancy period for 164 births where the mother received inpatient or outpatient psychiatric treatment during the pregnancy period**.^a^

Category of birth	Number of births	Average length of stay per birth (days)	Average no. of admissions per birth
Births involving any psychiatric hospitalization of mother during pregnancy period	119 (73%)	37	2.1
Births involving any psychiatric hospitalization of mother during pregnancy period and mother taking psychotropic medication	104 (83% of 119)	37	1.9
Births involving involuntary hospitalization of mother during pregnancy period	60 (37%)	36	1.4
Births involving involuntary hospitalization of mother during pregnancy period and mother taking psychotropic medication	51 (85% of 60)	36	1.4

*^a^Mothers who gave birth during 1980–1992 and had also been an inpatient at the State Psychiatric Hospital during 1966–1996*.

Table 3 shows medication intake, alcohol, and substance abuse across the pregnancy period. The number of mothers taking psychotropic medication was significantly reduced when the period 6 months before pregnancy was compared with the first trimester but there were no further significant changes in the following trimesters. Typical antipsychotics were the most common form of medication consumed. The medications prescribed for these mothers while they were in hospital included typical antipsychotics, tricyclic antidepressants, mood stabilizers, benzodiazepines, and hypnotics. Selective serotonin reuptake inhibitors (SSRIs) and Reversible Monoamine Oxidase Inhibitor Antidepressants were not available during the period of the study (17). The number of mothers using substances, which were mainly alcohol and cannabis, appeared to show a decline from the prepregnancy period to the end of the pregnancy. However, further inspection of the data indicates that the mothers using substances during the prepregnancy period were a different group to those using substances during pregnancy.

**Table 3:** **Medication intake and substance use by pregnancy period**.

Medication type	6 months before pregnancy		Trimester 1		Trimester 2		Trimester 3		Total	
					
	*N* = 158	%	*N* = 149	%	*N* = 149	%	*N* = 152	%	*N* = 164	%
Any medication	87	55	44**	30	53	36	53	35	132	80
Typical antipsychotic	79	50	36**	24	47	32	43	28	119	73
Antidepressant (not SSRI, RIMA)	12	8	4*	3	3	2	4	3	19	12
Mood stabilizer	24	15	9**	6	9	6	13	9	34	21
Benzodiazepine and anxiolytic/hypnotics	27	17	6**	4	6	4	11	7	39	24
Substance abuse (including alcohol and cannabis)	14	9	9	6	9	6	3	2	28	17
Alcohol abuse	9	6	5	3	6	4	3	2	18	11
Cannabis abuse	6	4	6	4	5	3	1	1	15	9

*p < 0.01, **p < 0.001 significant difference between value 6 months before pregnancy and at trimester 1.

*SSRI, selective serotonin reuptake inhibitor; RIMA, reversible inhibitors of monoamine oxidase A*.

## Discussion

In this descriptive study, we have documented the sociodemographic circumstances and the use of psychotropic medication and substances during pregnancy for women with severe mental illness who received psychiatric inpatient or outpatient services during the pregnancy period.

### Medication Use

The majority of the women who were hospitalized took psychotropic medication (80%), which is indicative of the severity of the disease, with antipsychotics recorded as the dominant medication category (73%) followed by mood stabilizers (21%), anxiolytic/hypnotics (16%), antidepressants (12%), and benzodiazepines (9%). Our data show fewer women taking medication during pregnancy than in the period immediately before pregnancy. This may be because the women are concerned about the potential effect of their medication on the developing fetus (18), or on the recommendation of their clinician, or because the severity of their illness declined during pregnancy. This finding of a decline in use is supported by a study by Toh et al. of 585,615 deliveries in the US to women aged 15–45 years during 2001–2007. Toh et al. found that 4,223 (0.72%) of the women were exposed to atypical antipsychotics and 548 to typical antipsychotics (0.09%) any time from 60 days before pregnancy until delivery (19). Of the women in Toh’s study taking atypical antipsychotics, the most common diagnosis was depression (63%) followed by bipolar disorder (43%) and schizophrenia (13%). Some 0.5% of women filled a prescription for atypical antipsychotics during the first trimester, 0.3% in the second trimester, and 0.2% in the third trimester. As with atypical antipsychotics, the prevalence of typical antipsychotic usage was greatest during the first trimester and then dropped in the last two semesters.

A similar study in Germany (2) on a cohort of 41,293 women who gave birth between June 2000 and May 2001 showed decreasing prescription rates from the period before pregnancy to the end of the pregnancy for antidepressants and antipsychotics, as did a Swedish study (4). Studies on the use of antidepressants during pregnancy indicate that the strongest decline in use is seen after the first trimester (3).

### Substance Use

Psychotic illness is a known risk factor for substance use (20, 21). Nine percent of those hospitalized in our study were recorded as using substances while pregnant. Five and a half percent of those hospitalized also used alcohol while pregnant. This compares with data from the United States (22) in 2002–2003, which showed that 4.7% of pregnant women reported any illicit substance use in the previous 30 days and 10% reported similar alcohol use. The comparable rate of alcohol consumption in non-pregnant women was 53.0%. Use of any substance including cigarettes was 63.9% in non-pregnant women and 25.8% in pregnant women. The study (22) reported reduced use of substances in the second and third trimesters as opposed to the first trimester and that the odds ratio for recent substance abuse among those with possible current psychopathology was 2.83. It is likely that the level of substance abuse is underreported in our study as it has only been recorded when the mother was in hospital.

### Suicide and Non-Suicidal Self-Injury

The risk of suicide and non-suicidal self-injury are reduced during pregnancy in the general population (23, 24). As we do not have data for before and after pregnancy in our study, it is not possible to see whether our results were significantly lower or higher during pregnancy. This is an area requiring further research.

### Demography

The demographic profile of the women shows little difference between those taking and not taking psychotropic medication. Differences between those receiving and not receiving treatment, however, may be associated with the location of the Statewide psychiatric hospital, which is in the metropolitan area and thus less accessible to those from regional and remote areas. As the majority of the aboriginal population live in rural and remote areas, this is likely to be why aboriginal mothers have a lower treatment rate. Higher treatment rates for single mothers are likely to be associated with a low level of support available at home (25). The higher admission rate for older mothers is consistent with the higher admission rate seen in women aged 35–44 for mental illness (26).

### Advantages and Limitations of This Study

There are a number of potential limitations to this study. The data are from the period 1980–1992 when the newer forms of antipsychotics and antidepressants were not widely available: atypical antipsychotics and SSRIs were introduced in the early nineties (17). Data were collected on substance use but this did not include smoking despite its documented association with negative pregnancy outcomes. It is likely that substance use has been underreported, particularly in lighter users, due to its social undesirability. This would result in bias toward the null hypothesis. The strength of the study comes from its methodology which combines record linkage to administrative midwives and psychiatric records with case notes review. This, in turn, has ensured that detailed information is available for each case, that the study is not reliant on retrospective recall and that the results are still relevant to current practice. Moreover, despite the small sample size, we have also been able to document a reduction in medication use during the pregnancy period compared to 6 months before pregnancy.

## Conclusion

This study on a cohort of 428 women of whom 164 received inpatient or outpatient psychiatric care for severe mental illness during the course of their pregnancy found that despite the severity of their illness, the women demonstrate a pattern of decreased use of psychotropic medication use from the period before pregnancy to the first trimester of pregnancy. Our study cannot provide data on the reason for this change, but we speculate that it may reflect motivation by the treating clinician, the mother, or both to maximize outcomes for the babies. However, of note, recently published clinical guidelines (27) for the management of schizophrenia and related disorders highlight the lack of evidence-based information about the safety of antipsychotic medicines during pregnancy. These data are essential to inform medication management for women with severe mental illness during pregnancy, and much more research in this area is warranted to optimize benefits for both mother and child. These results highlight the importance of women with severe mental illness receiving regular ongoing monitoring and support from their psychiatrist during pregnancy regarding the level of medication required, substance use, and with regard to non-suicidal self-injury and attempted suicide.

## Ethics Statement

The study had University of Western Australia Human Research Ethics Committee approval as well as specific approvals from the individual inpatient and outpatient mental health services at which the clinical records were held. Participant consent was not required for this study of medical records. Requirement for consent was waived on the basis of the study being low risk, the benefits from the research justified any risks of harm associated with not seeking consent and privacy and confidentiality were protected by meeting the required standards for data storage and security.

## Author Contributions

KB analyzed the data and drafted the paper, AJ was responsible for conception and design of the study and coauthored the paper, VM was involved in the study design, supervised local data collection, performed overall data management, supervised the data analysis, and coauthored the paper. JD and JG conducted the case note review and coauthored the paper. All the authors read and approved the final manuscript.

## Conflict of Interest Statement

The authors declare that the research was conducted in the absence of any commercial or financial relationships that could be construed as a potential conflict of interest.

## References

[1] Timmermans S Bonsel GJ Steegers-Theunissen RP Mackenbach JP Steyerberg EW Raat H Individual accumulation of heterogeneous risks explains perinatal inequalities within deprived neighbourhoods. Eur J Epidemiol (2011) 26:165–80. 10.1007/s10654-010-9542-5 21203801 PMC3043261

[2] Egen-Lappe V Hasford J. Drug prescription in pregnancy: analysis of a large statutory sickness fund population. Eur J Clin Pharmacol (2004) 60:659–66. 10.1007/s00228-004-0817-1 15480609

[3] Kallen B Nilsson E Otterblad Olausson P Antidepressant use during pregnancy: compariosn of data obtained from a prescription register and from antenatal care records. Eur J Clin Pharmacol (2011) 67:839–45. 10.1007/s00228-011-1021-8 21387167

[4] Stephansson O Granath F Svensson T Haglund B Ekbom A Kieler H. Drug use during pregnancy in Sweden – assessed by the prescribed drug register and the medical birth register. Clin Epidemiol (2011) 3:43–50. 10.2147/CLEP.S16305 21386973 PMC3046184

[5] McNeil TF Kaij L Malmquist-Larsson A. Women with nonorganic psychosis: pregnancy’s effect on mental health during pregnancy. Acta Psychiatr Scand (1984) 70:140–8. 6485847 10.1111/j.1600-0447.1984.tb01191.x

[6] Cohen LS Altshuler LL Harlow BL Nonacs R Newport DJ Viguera AC Relapse of major depression during pregnancy in women who maintain or discontinue antidepressant treatment. JAMA (2006) 295:499–507. 10.1001/jama.295.5.499 16449615

[7] Austin MP Kildea S Sullivan E. Maternal mortality and psychiatric morbidity in the perinatal period: challenges and opportunities for prevention in the Australian setting. Med J Aust (2007) 186:364–7. 17407434 10.5694/j.1326-5377.2007.tb00940.x

[8] Austin MP Mitchell PB. Psychotropic medications in pregnant women: treatment dilemmas. Med J Aust (1998) 169:428–31. 9830392 10.5694/j.1326-5377.1998.tb126837.x

[9] King-Hele S Webb R Mortensen PB Appleby L Pickles A Abel KM Risk of stillbirth and neonatal death linked with maternal mental illness: a national cohort study. Arch Dis Child Fetal Neonatal Ed (2009) 94:F105–10. 10.1136/adc.2007.135459 19000999

[10] Gentile S. Drug treatment for mood disorders in pregnancy. Curr Opin Psychiatry (2011) 24:34–40. 10.1097/YCO.0b013e3283413451 21088587

[11] Boden R Lundgren M Brandt L Reutfors J Andersen M Kieler H. Risks of adverse pregnancy and birth outcomes in women treated or not treated with mood stabilisers for bipolar disorder: population based cohort study. BMJ (2012) 345:e7085. 10.1136/bmj.e7085 23137820 PMC3493986

[12] World Health Organization. International Statistical Classification of Diseases and Related Health Problems, Ninth Revision (ICD-9). Geneva: World Health Organisation (1977).

[13] Castle DJ Jablensky A McGrath JJ Carr V Morgan V Waterreus A The diagnostic interview for psychoses (DIP): development, reliability and applications. Psychol Med (2006) 36:69–80. 10.1017/S0033291705005969 16194284

[14] Jablensky AV Morgan V Zubrick SR Bower C Yellachich LA Pregnancy, delivery, and neonatal complications in a population cohort of women with schizophrenia and major affective disorders. Am J Psychiatry (2005) 162:79–91. 10.1176/appi.ajp.162.1.79 15625205

[15] Australian Bureau of Statistics. An Introduction to Socio-Economic Indexes for Areas. 2006. Canberra: Commonwealth of Australia (2008).

[16] Glover J Tennant S Remote Areas Statistical Geography in Australia. Notes on the Accessibility/Remoteness Index for Australia (ARIA+ version). Adelaide: Public Health Information Development Unit (2003).

[17] Mant A Rendle VA Hall WD Mitchell PB Montgomery WS McManus PR Making new choices about antidepressants in Australia: the long view 1975-2002. Med J Aust (2004) 181:S21–4. 15462638 10.5694/j.1326-5377.2004.tb06350.x

[18] Viguera AC Cohen LS Bouffard S Whitfield TH Baldessarini RJ. Reproductive decisions by women with bipolar disorder after prepregnancy psychiatric consultation. Am J Psychiatry (2002) 159:2102–4. 10.1176/appi.ajp.159.12.2102 12450965

[19] Toh S Li Q Cheetham T Cooper W Davis R Dublin S Prevalence and trends in the use of antipsychotic medications during pregnancy in the US, 2001-2007: a population-based study of 585,615 deliveries. Arch Womens Ment Health (2013) 16:149–57. 10.1007/s00737-013-0330-6 23389622 PMC3715880

[20] Jablensky A McGrath JJ Herrman H Castle DJ Gureje O Morgan VA People Living with Psychotic Illness: An Australian Study 1997-98. Canberra: National Survey of Mental Health and Wellbeing, Commonwealth of Australia, National Mental Health Strategy (1999).

[21] Morgan VA Waterreus A Jablensky AV Mackinnon A McGrath JJ Carr V People Living with Psychotic Illness 2010. Report on the Second Australian National Survey. Canberra: Australian Government (2011). 10.1177/0004867412449877 22696547

[22] Havens JR Simmons LA Shannon LM Hansen WF. Factors associated with substance use during pregnancy: results from a national sample. Drug Alcohol Depend (2009) 99:89–95. 10.1016/j.drugalcdep.2008.07.010 18778900

[23] Appleby L. Suicide during pregnancy and in the first postnatal year. BMJ (1991) 302:137–40. 1995132 10.1136/bmj.302.6769.137 PMC1668816

[24] Lindahl V Pearson JL Colpe L. Prevalence of suicidality during pregnancy and the postpartum. Arch Womens Ment Health (2005) 8:77–87. 10.1007/s00737-005-0080-1 15883651

[25] Rabinowitz J Mark M Popper M Slyuzberg M Munitz H. Predicting revolving-door patients in a 9-year national sample. Soc Psychiatry Psychiatr Epidemiol (1995) 30:65–72. 7754418 10.1007/BF00794944

[26] Australian Institute of Health and Welfare. Specialised Admitted Mental Health Care Patient Characteristics, Mental Health Services in Australia. Admitted Patient Mental Health-Related Care. Canberra: Australian Government (2010).

[27] Galletly C Castle D Dark F Humberstone V Jablensky A Killackey E Royal Australian and New Zealand College of Psychiatrists clinical practice guidelines for the management of schizophrenia and related disorders. Aust New Zeal J Psychiatr (2016) 50:410–72. 10.1177/0004867416641195 27106681

